# Immune Responses in Laying Hens after an Infectious Bronchitis Vaccination of Pullets: A Comparison of Two Vaccination Strategies

**DOI:** 10.3390/vaccines9050531

**Published:** 2021-05-20

**Authors:** Sabrina M. Buharideen, Mohamed S. H. Hassan, Shahnas M. Najimudeen, Dongyan Niu, Markus Czub, Susantha Gomis, Mohamed Faizal Abdul-Careem

**Affiliations:** 1 Faculty of Veterinary Medicine, University of Calgary, Health Research Innovation Center 2C53, 3330 Hospital Drive NW, Calgary, AB T2N 4N1, Canada; sabrina.buharideen@ucalgary.ca (S.M.B.); msh.hassan@ucalgary.ca (M.S.H.H.); fathimashahnas.moham@ucalgary.ca (S.M.N.); dongyan.niu@ucalgary.ca (D.N.); m.czub@ucalgary.ca (M.C.); 2 Department of Veterinary Pathology, Western College of Veterinary Medicine, 52, Campus Drive, University of Saskatchewan, Saskatoon, SK S7N 5B4, Canada; smg127@mail.usask.ca

**Keywords:** infectious bronchitis virus, laying hen, infectious bronchitis vaccine, cell-mediated immune response, antibody-mediated immune response

## Abstract

For decades, vaccinations have been used to limit infectious bronchitis (IB) in both the broiler and layer industries. Depending on the geographical area, live attenuated vaccines are used either alone or in combination with inactivated vaccines to control infectious bronchitis virus (IBV) infections. It has been shown that administering inactivated vaccines preceded by priming with live attenuated vaccines in pullets protects laying hens against IB. However, the immunological basis of this protective response has not been adequately investigated. The objective of the study was to compare two vaccination strategies adapted by the Canadian poultry industry in terms of their ability to systemically induce an adequate immune response in IBV-impacted tissues in laying hens. The first vaccination strategy (only live attenuated IB vaccines) and second vaccination strategy (live attenuated and inactivated IB vaccines) were applied. Serum anti-IBV antibodies were measured at two time points, i.e., 3 weeks and 10 weeks post last vaccination. The recruitment of T cell subsets (i.e., CD4+ and CD8+ T cells), and the interferon (IFN)-γ mRNA expression were measured at 10 weeks post last vaccination. We observed that vaccination strategy 2 induced significantly higher serum anti-IBV antibody responses that were capable of neutralizing an IBV Mass variant associated with a flock history of shell-less egg production better than a Delmarva (DMV)1639 variant, as well as a significantly higher IFN-γ mRNA expression in the lungs, kidneys, and oviduct. We also observed that both vaccination strategies recruited CD4+ T cells as well as CD8+ T cells to the examined tissues at various extents. Our findings indicate that vaccination strategy 2 induces better systemic and local host responses in laying hens.

## 1. Introduction

Infectious bronchitis virus (IBV) causes a globally prevalent disease, namely, infectious bronchitis (IB), that affects chickens [1]. IBV is a gamma coronavirus that belongs to the *Coronaviridae* family [2,3]. It contains a linear single-stranded (ss), ribonucleic acid (RNA) genome. The genome is approximately 27.6 kilobases (kb) long and codes for large replicase polyprotein, as well as structural proteins (i.e., spike (S) glycoprotein, membrane (M) protein, nucleocapsid (N) protein, and small envelope protein (E) [2,4]. IB has clinical and pathological consequences in multiple body systems such as respiratory, renal, and reproductive tracts [5,6,7,8]. Initially, IBV targets the upper respiratory epithelial cells and leads to respiratory clinical manifestations [5,9]. In the kidneys, the ciliated epithelial cells of nephrons are infected by nephropathogenic IBV strains, which results in clinical signs such as weight loss, watery droppings, and an increased incidence of mortality [7,8,10,11]. In the reproductive tract, IBV targets the cells of oviducts in chickens, potentially causing hens to lay eggs with shell abnormalities and thus decreasing egg production [6,12,13].

The distribution of IBV serotypes in Canada varies depending on geographical location. In eastern Canada, the most common IBV serotypes include Delmarva (DMV/1639) and 4/91 IBV [14,15]. In western Canada, the circulating IBV strains are mainly Massachusetts (Mass) and Connecticut (Conn) variants [13]. Due to this variation in IBV serotype distribution, vaccination protocols adapted by the table-egg layer industry vary across the country since the vaccine-induced immunity is serotype specific [16]. Although a number of novel vaccines has been experimentally tested, only live attenuated and inactivated vaccines are currently commercially available [16]. The live attenuated vaccine, Mass strain M41, was developed in the 1950s to control IB in poultry operations [17,18,19]. These live attenuated vaccines are administered via drinking water, coarse spray, or as eye drops [20,21]. Moreover, the booster vaccination is given with the same serotype or in combination with a different serotype in the live vaccine [22]. Inactivated IB vaccines are given via an intramuscular route routinely in layer chickens and produced by the chemical inactivation of the live IBV and suspended in the aqueous phase of an oil adjuvant emulsion to enhance the immune response. However, weak immune responses were induced when given alone [23]. High levels of antigen and priming using live attenuated vaccines are typically needed for inactivated IB vaccines in order to produce effective immune responses [24]. Although the poultry industry relies on vaccinations, IBV infection often occurs in vaccinated and non-vaccinated flocks, causing severe losses over the last few years. The vaccination failure may be related to the frequent emergence of new variants or serotypes of IBV [12,25,26,27]. Therefore, it is important to characterize the prevalent serotypes of IBV in a region and combine different serotypes of IB vaccines to induce cross protection [28].

Only the live attenuated vaccines—Mass and Conn serotypes—and the inactivated vaccines—Mass and Arkansas (Ark) serotypes—are available in Canada. Consequently, these live attenuated and inactivated vaccines are given in various combinations to control IB in poultry operations [29]. Commercial table-egg layers in Canada are vaccinated starting from 1 day to 2 weeks, and every 4–6 weeks until 16 weeks of age. Apart from the vaccination of pre-lay pullets, the laying hens are not vaccinated in Canada. Some producers rely only on various blends of live attenuated vaccines given from 3 weeks of age, and others rely on an additional inactivated vaccine given intramuscularly before the laying period. It has been shown that the vaccination strategies that use live attenuated plus inactivated vaccines induce 90% or more protection when compared to the use of live attenuated vaccines alone [30,31,32]. Further, effective protection against nephropathogenic BR-I IBV was achieved by antibody- and cell-mediated immune response induced by the combination of a live attenuated vaccine of the Mass serotype and an inactivated vaccine that has the BR-I IBV strain [33]. It is not known if host responses, particularly mucosal immune responses generated by these different vaccination strategies, are different in laying hens. Moreover, since certain strains of IBV may infect and replicate within tissues such as the kidneys and reproductive tissues (magnum, uterus, and isthmus) other than the respiratory tract, it is crucial to evaluate whether immunological responses induced by the vaccine can be valid in the non-respiratory system [6,7,34,35]. Therefore, the objective of this study was to compare the host immune responses induced by two different IB vaccination strategies employed during the pullet growing period in the lungs, kidneys, and reproductive tissues of egg-laying hens.

## 2. Materials and Methods

### 2.1. Animals

Two-week-old White Leghorn specific pathogen free (SPF) layer chickens were purchased from the Canadian Food Inspection Agency (CFIA) (Ottawa, ON, Canada) and housed at the University of Calgary’s Veterinary Science Research Station (VSRS). The work described in the manuscript was approved by the University of Calgary’s Health Science Animal Care Committee (HSACC) (Animal use protocol number: AC19-0113).

### 2.2. Vaccines

Live attenuated IB vaccines contained the Mass serotype (Merial INC, Athens, GA, USA), the Mass and Conn serotypes (Zoetis Inc, Kalamazoo, MI, USA), and the inactivated IB vaccine contained the Mass serotype (Merck Animal Health, Division of Intervet. INC, Omaha, NE, USA). The vaccines were stored at 4 °C.

### 2.3. Experimental Design

The two-week-old SPF chickens were assigned to groups as indicated in Table 1. At 3 weeks of age, Group 1 received live attenuated IB vaccines (vaccination strategy 1), whereas group 2 received live attenuated IB vaccines followed by an inactivated IB vaccine (vaccination strategy 2). Group 3 was maintained as the mock vaccinated control. The vaccination that used live attenuated vaccines was completed following the manufacturer’s instructions via eye drops (30 µL per chicken). The eye drop method was preferred over spray and drinking water routes in order to deliver an exact dose of the vaccine uniformly within the vaccinated animals. The inactivated IB vaccine was also given intramuscularly per the manufacturer’s instructions (0.5 mL per chicken). At 3 weeks post last vaccination, 3 mL of blood samples were collected from the chicken wing vein from all groups, and the serum anti-IBV antibody titers were determined as prescribed by the manufacturer via a commercial enzyme-linked immunosorbent assay (ELISA) kit (IDEXX Laboratories, Westbrook, ME, USA) that uses inactivated IBV virions as coating antigens. At 10 weeks post last vaccination (peak of lay), the chickens in all groups were euthanized. We chose to examine the tissues at peak of lay since IB outbreaks in layers are common during peak of lay [36]. The blood samples were collected to measure the serum anti-IBV antibody titers and to conduct the virus neutralization assay against an IBV Mass variant linked to shell-less egg production in chickens and IBV DMV1639 linked to false layer syndrome [13,37]. Tissue samples from the lungs, kidneys, and reproductive tissues (magnum, isthmus, and uterus) were collected in RNASave (Biological Industries, Frogga Bio, Toronto, ON, Canada) and the optimum cutting temperature (OCT) medium (Tissue-Tek^®^, Sakura Finetek USA Inc., Torrance, CA, USA) in order to study interferon (IFN)-γ mRNA expression and immune cell recruitments, respectively. The CD4+ and CD8+ T cells were detected using the immunofluorescent technique. The whole spleen was collected in a cold Hank’s balanced salt solution (HBSS) to isolate mononuclear cells. The mononuclear cells were isolated to conduct a flow cytometry technique needed to quantify CD4+ and CD8+ T cells.

### 2.4. Techniques

#### 2.4.1. Enzyme-Linked Immunosorbent Assay (ELISA)

The serum IBV antibody titers were measured using the IDEXX IBV antibody ELISA kit (IDEXX IBV Laboratories, Westbrook, ME, USA). Diluted (1:500) serum samples were used in the assay. The wells in the 96 well plate were then coated with samples or negative and positive controls following the manufacturer’s instructions. The optical density (OD) at 650 nanometers (nm) was measured using a microplate reader (Molecular Devices, Sunnyvale, NS, Canada). The titers were calculated using the provided equation given in the manual guidelines (Log10 titer = 1.09 (log10 Sample/Positive) + 3.36). The serum IBV antibody titers ≥ 396 were considered positive.

#### 2.4.2. Virus Neutralization (VN) Assay

The β-virus neutralization (VN) procedure, with a fixed concentration of a virus (IBV Mass variant linked to shell-less egg production and DMV/1639 linked egg production problems and false layer syndrome) [13,37] and serial dilutions of serum, was carried out. Serum collected from the chickens that received vaccination strategy 1 and 2 was pooled separately and inactivated at 56 °C for 30 min before being used in the VN assay. Twofold serial dilutions of serum were mixed with an equal volume of virus dilution containing 200 EID_50_ in 0.1 mL and incubated for 1 h at room temperature. Each serum–virus mixture was then inoculated in 5 SPF 9-day old chicken embryonated eggs via the intra-allantoic route. Non-specific chicken embryo mortality was excluded 24 h after inoculation, and eggs were incubated for 6 more days to evaluate the presence of specific lesions of IBV infection such as stunting and curling of the embryo. End-points corresponded to the serum dilutions that neutralized 50% of the virus. End-point titers were calculated by the Spearman–Kärber method [38].

#### 2.4.3. Spleen Mononuclear Cell Isolation

Spleens were dissected and maintained in an ice-cold Hank’s balanced salt solution (HBSS, Life Technologies Corporation, Grand Island, NY, USA), then rinsed several times to ensure they were free of blood. Spleens were homogenized using a sterile syringe bottom and filtered through a 70 micrometer (μM) cell strainer (Life Sciences, Durham, NC, USA). The filtered spleen cells were spun at 1000 rounds per minute (rpm) for 10 min (4 °C). The pellet was suspended in 4 mL 10% of a Roswell Park Memorial Institute Medium 1640 (RPMI) (Life Technologies Corporation, Grand Island, NY, USA). The cell suspension (4 mL) was carefully layered onto 3 mL of the Ficoll-Paque solution (GE Healthcare Bio-Sciences AB, SE, Uppsala, Sweden) in a 15 mL tube at room temperature. The tubes with layered cells were spun at 1890 rpm (18 to 20 °C) for 40 min. The cloudy layer consisting of mononuclear cells was collected in a separate 15 mL tube and washed with HBSS. Cells were suspended in a 10% RPM1 medium consisting of 1% L-glutamine (Life Technologies Corporation, Grand Island, NY, USA), 1% penicillin–streptomycin (Life Technologies Corporation, Grand Island, NY, USA), and 10% fetal bovine serum (FBS) (Life Technologies Corporation, Grand Island, NY, USA). The harvested single-cell suspension was stained with trypan blue (Life Technology Corporation, Grand Island, NY, USA) and counted using a hematocytometer (Hausser Scientific, Horsham, PA, USA).

#### 2.4.4. Flow Cytometry Technique

Mononuclear cells (2 × 10^6^ cells per well) were stained on 96-well U-bottom microtiter plates (Nunc A/S, DK, Roskilde, Denmark). The cells were washed in 1% bovine serum albumin (BSA) (Sigma-Aldrich Co, St, Louis, MO, USA), which was made in a phosphate buffered saline (PBS) (Life Technologies Corporation, Grand Island, NY, USA). The wash buffer was discarded following centrifugation at 150 rpm for 5 min (4 °C). The cells were suspended in 0.2% chicken serum for 15 min (diluted in 1% BSA) for Fc blocking. The cells were then spun as described above and the pellets containing cells were suspended in the dark with CD8 (anti-chicken mouse CD8 alpha conjugated to FITC) (Southern Biotech, Birmingham, AL, USA) and CD4 (anti-chicken mouse CD4 conjugated to PE (Southern Biotech, Birmingham, AL, USA) monoclonal antibodies on ice for 30 min. At the end of the 30 min, the stained mononuclear cells were washed with 1% BSA and fixed with 1% paraformaldehyde (Electron Microscopy Sciences, Hatfield, PA, USA). The samples were analyzed at the Flow Cytometry Core Facility, University of Calgary.

#### 2.4.5. Immunofluorescent Assay for CD4+ and CD8+ T Cells

The OCT-preserved frozen tissues were cut at 5 µM using a cryotome (Leica Biosystems Inc., Concord, ON, Canada) and adhered to positively charged slides (VWR, Mississauga, ON, Canada). They were then preserved at −20 °C until further processing. Before staining for CD4+ T cells, the tissues were air-dried for 20 min on the bench and dipped in cold acetone for 5 min. The tissues were then blocked with avidin and biotin (Vector Laboratories, Inc., Burlingame, CA, USA) for 15 min, followed by blocking with 5% goat serum in Trizma buffered saline (TBS) for 30 min. After that, the sections were soaked with a 1:200 dilution of unconjugated mouse monoclonal antibody specific to chicken CD4+ T cells (Southern Biotech, Birmingham, AL, USA) in 5% goat serum and placed in a humidified chamber for 30 min. Next, the sections were incubated with a 1:250 dilution of biotinylated goat anti-mouse IgG (H + L) (Southern Biotech, Birmingham, Alabama, USA), followed by a 15:1000 dilution of DyLight^®^ 488 streptavidin (Vector Laboratories Inc., Burlingame, CA, USA), each for 30 min. After each step, the tissue sections were washed with a TBS-T buffer (Tris-buffered saline with 0.1% Tween 20) (2 × 5 min) and PBS (1 × 5 min).

To stain the sections for CD8+ T cells, tissue sections were air-dried for 20 min on the bench and left in ice cold acetone for 5 min. Then, the tissue sections were treated with 5% goat serum for 30 min. After the incubation, the sections were covered with unconjugated mouse monoclonal antibody specific for chicken CD8α (Southern Biotech, Birmingham, Alabama, USA) (1:200 dilution). The incubation with a secondary antibody—i.e., goat anti-mouse IgG (H + L) conjugated with Dylight^®^ 550 (1:500 dilution, Bethyl Laboratories Inc., Montgomery, TX, USA)—was performed for 1 h. Following each step, the tissue section was washed with TBST (2 × 5 min) and PBS (1 × 5 min). Finally, the slides stained for T cell subsets (CD4+ and CD8+ T cells) were mounted with a Vectashield^®^ mounting medium via 4′,6-diamidino-2-phenylindole (DAPI) (Vector Laboratories Inc., Burlingame, CA, USA) before being cover slipped.

#### 2.4.6. RNA Extraction and Complementary (c) DNA Synthesis

RNA was extracted from the lungs, kidneys, and reproductive tract tissues using Trizol reagent (Invitrogen Canada Inc., Burlington, ON, Canada) following the manufacturer’s instruction. Briefly, the tissues (50 mg) were homogenized using a microtube homogenizer (Ambion, Invitrogen Canada Inc, Burlington, ON, Canada) in 1 mL of Trizol reagent. Then, 200 μL of chloroform was added. The tubes were spun at 12,000× *g* for 15 min at 4 °C. They were then incubated for 10 min at room temperature following the transfer of the upper phase of the solution containing RNA into a separate tube and adding isopropanol (500 μL). The tubes were spun again at 12,000× *g* for 11 min (4 °C) and the supernatant was carefully discarded. Next, 1 mL of 75% ethanol was added and the tubes were spun at 7500× *g* for 5 min. After the supplement was discarded, the pellet was air-dried, suspended in 20 μL of RNA free water, and incubated for 10 min at 56 °C. The quantity of RNA was measured using a Nanodrop 1000 spectrophotometer (Thermo Scientific, Wilmington, DE, USA). The extracted RNA was used to synthesize cDNA using RT random primers (high capacity cDNA reverse transcriptase kit, Invitrogen Life Technologies, Carlsbad, CA, USA) according to the manufacturer’s guidelines.

#### 2.4.7. Real-Time Quantitative Polymerase Chain Reaction (qPCR) Assay

The CFX 96-c1000 Thermocycler (Bio- Rad Laboratories, Mississauga, ON, Canada) was used to quantify INF-γ mRNA expression. The INF-γ gene expression was quantified relative to the mRNA expression of the housekeeping gene, β-actin. The qPCR assays for both the β- actin and INF-γ genes were run on the same plate. Fast SYBR^®^ Green Master Mix (Invitrogen, Burlington, ON, Canada) was used. The primers for the INF-γ gene (F-ACACTGACAAGTCAAAGCCGCACA, R-AGTCGTTCATCGGGACCTTGGC) [39] and primers for the β-actin gene (F-CAACACAGTGCTGTCTGGTGGTA, R-ATCGTACTCCTGCTTGCTGATCC) [39] were used in each reaction. Each reaction volume consisted of 10 µL of SYBR Green master mix, 200 ng of cDNA of respective samples as a template, and 0.5 µL of forward and reverse specific primers that targeted the genes. The qPCR conditions were 95 °C for 20 s of pre-incubation and 95 °C for 3 s. For 40 amplification cycles, it was 60 °C for 30 s. Then, 95 °C and 65 °C with a 0.5 °C rise in temperature every 5 s was used for the melting curve analysis.

### 2.5. Data Analyses

The Pfaffl method was used to determine the relative IFN-γ expression via the change in the IFN-γ mRNA expression relative to the housekeeping gene (β-actin) in the tissues [40]. The CD4+ and CD8+ T cells were quantified, capturing 5 areas of each tissue at 20x magnification using an epifluorescent microscope (Olympus IX51, Center Valley, Pensylvania, USA). Moreover, nuclear stained (DAPI positive) areas of each tissue were captured. Using the Image-J software (National Institute of Health, Bethesda, Maryland, USA) the density of the cells in each area was quantified. The Kruskal–Wallis test and Dunn’s multiple comparisons test were used to analyze the IFN-γ mRNA expression, anti-IBV antibody titers, and CD4+ and CD8+ T cells in tissues. The significance was set at *p* ≤ 0.05. All statistical analyses were performed in the GraphPad Prism 5 Software (GraphPad Prism 5 Software, La Jolla, CA, USA).

## 3. Results

### 3.1. Serum Anti-IBV Antibody Response

**Figure 1:**
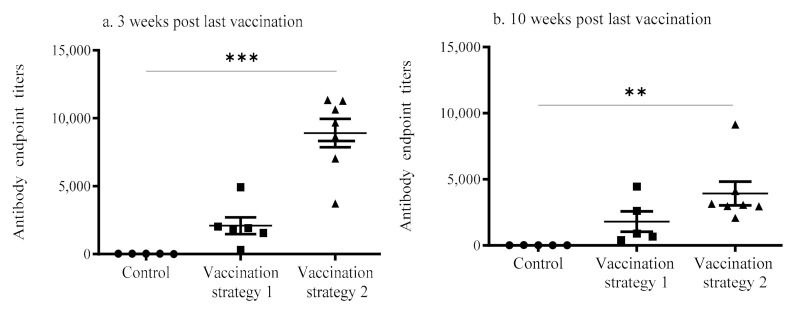
Serum anti-IBV antibody titers of the vaccinated (vaccination strategies 1 and 2) and mock vaccinated groups at 3 weeks (**a**) and 10 weeks (**b**) post last vaccination. The blood samples were collected and the enzyme-linked immunosorbent assay (ELISA) was used to determine the serum anti-IBV antibody titers. The data were analyzed statistically using the Kruskal–Wallis test followed by Dunn’s multiple comparison test. The statistical significance is indicated as * (** = significant at *p* < 0.001, *** = significant at *p* < 0.0001).

The anti-IBV antibody concentrations in the vaccinated (groups 1 and 2) and mock vaccinated groups are depicted in Figure 1. At 3 weeks post last vaccination, the anti-IBV antibody concentrations for chickens given the second vaccination strategy were significantly higher (*p* < 0.0001) when compared to those in the mock vaccinated controls (Figure 1a). At 10 weeks post last vaccination, the anti-IBV antibody concentrations of chickens given the second vaccination strategy remained significantly higher (*p* < 0.05) compared to mock vaccinated controls (Figure 1b). By contrast, the serum antibody titers of the chickens given in the first vaccination strategy did not statistically differ (*p* > 0.05) from those of the mock vaccinated controls at both time points. There was no statistically significant difference evident between vaccination strategies 1 and 2 (*p* > 0.05).

### 3.2. Virus Neutralization

The VN assay data showed that the serum of the chickens that received vaccination strategy 2 collected at 3 weeks post last vaccination was able to neutralize the IBV Mass variant linked to shell-less egg production (the titer was >1024) better when compared to the serum of chickens that received vaccination strategy 1 collected at 3 weeks post last vaccination (the titer was ≤64). However, when we used DMV/1639 IBV, we found the neutralization titer of the vaccination strategy 2 serum was ≤128 and for the vaccination strategy 1 serum, the neutralization titer was ≤8.

### 3.3. Spleen CD4+ and CD8+ T Cells

**Figure 2:**
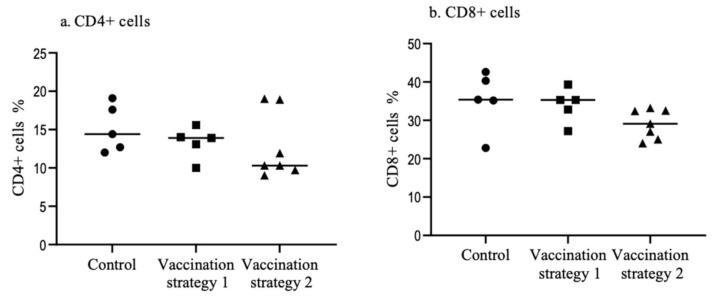
Percentage of CD4+ T cells (**a**) and CD8+ T cells (**b**) in the spleen of the vaccinated (vaccination strategies 1 and 2) and mock vaccinated groups. The spleens were collected at 10 weeks post last vaccination. Mononuclear cells were isolated and stained using the PE conjugated anti-chicken CD4 and FITC conjugated anti-chicken CD8 monoclonal antibodies for flow cytometry analyses. The data were analyzed statistically using the Kruskal–Wallis test followed by Dunn’s multiple comparison test.

At 10 weeks post last vaccination, CD4+ and CD8+ T cell populations were not different (*p* > 0.05) between vaccinated (vaccination strategies 1 and 2) and mock vaccinated controls (Figure 2).

### 3.4. CD4+ and CD8+ T Cell Recruitments in the Lungs, Kidneys, and Reproductive Tract Tissues

**Figure 3:**
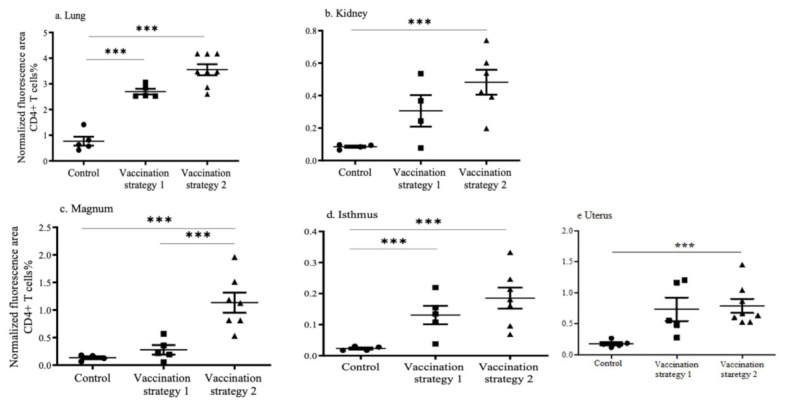
Recruitment of CD4+ T cells in tissues targeted by IBV following vaccination or mock vaccination. The CD4+ T cells in the lungs (**a**), kidneys (**b**), magnum (**c**), isthmus (**d**), and uterus (**e**) are illustrated. The cryopreserved tissues were sectioned and immunoassayed using unconjugated monoclonal antibodies directed against chicken CD4. Positive signals were quantified and normalized with nuclear stained areas. Statistical analysis was performed using the Kruskal–Wallis test, which was followed by Dunn’s multiple comparison test. The statistical significance is indicated as (*** = significant at *p* < 0.0001).

**Figure 4:**
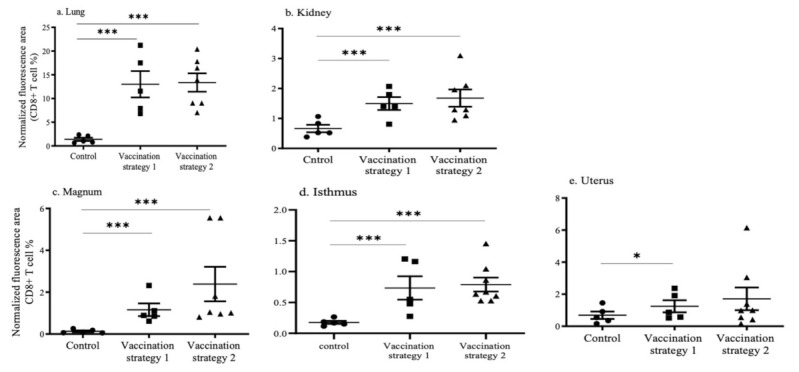
Recruitment of CD8+ T cells in tissues targeted by IBV following vaccination or mock vaccination. The CD8+ T cells in the lungs (**a**), kidneys (**b**), magnum (**c**), isthmus (**d**), and uterus (**e**) are illustrated. The cryopreserved tissues were sectioned and immunoassayed using monoclonal antibodies directed against chicken CD8. Positive signals were quantified and normalized with nuclear stained areas. The statistical analysis was performed using the Kruskal–Wallis test, which was followed by Dunn’s multiple comparison test. The statistical significance is indicated as (* = significant at *p* < 0.05, *** = significant at *p* < 0.0001).

Recruitments of CD4+ and CD+8 T cells in the lungs, kidneys, and reproductive tissues (magnum, isthmus, and uterus) in vaccinated and mock vaccinated chickens were quantified using the immunofluorescent technique (Figure 3 and Figure 4). The representative images of the CD4+ and CD8+ T cells in the lungs, kidney, and reproductive tissues are shown in Supplementary Figures S1 and S2.

In the lungs, the recruitment of CD4+ and CD8+ T cells was higher (*p* < 0.0001) in chickens vaccinated with vaccination strategies 1 and 2 when compared to mock vaccinated chickens (Figure 3a and Figure 4a). The recruitment of CD4+ and CD8+ T cells in the lungs of vaccination strategies 1 and 2 groups was not different (*p* > 0.05). In the kidneys, the recruitment of CD4+ T cells in chickens that received vaccination strategy 2 was higher (*p* < 0.0001) than the mock vaccinated controls (Figure 3b). The recruitment of CD8+ T cells in the kidneys of chickens who received vaccination strategies 1 and 2 was higher (*p* < 0.0001) than the mock vaccinated controls (Figure 4b).

In the reproductive tissues, the recruitment of CD4+ and CD8+ T cells in the magnum, isthmus, and uterus of the chickens who received vaccination strategy 2 was significantly higher (*p* < 0.0001) than the mock vaccinated controls (Figure 3c–e and Figure 4c–e). In the magnum, the recruitment of CD4+ T cells in chickens who received vaccination strategy 2 was higher (*p* < 0.0001) than those who received vaccination strategy 1 (Figure 3c). The recruitment of CD4+ T cells in the isthmus of chickens who received vaccination strategy 1 was higher (*p* < 0.0001) than the mock vaccinated controls (Figure 3d). The CD8+ T cell numbers in the reproductive tract are illustrated in Figure 4c–e. The recruitment of CD8+ T cells in the magnum and the isthmus of chickens who received vaccination strategy 2 was higher (*p* < 0.0001) than the mock vaccinated controls. The recruitment of CD8+ T cells in the isthmus and the magnum of chickens that received vaccination strategy 1 was also significantly higher (*p* < 0.0001) than the mock vaccinated controls.

### 3.5. INF-γ mRNA Expression

**Figure 5:**
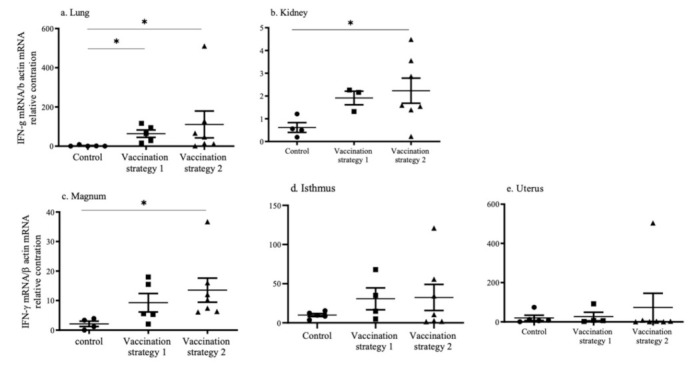
The INF-γ mRNA expression was measured in the lungs (**a**), kidneys (**b**), magnum (**c**), isthmus (**d**) and uterus (**e**) in vaccinated and mock vaccinated control chickens. The RNA was extracted from homogenized tissues and reverse transcribed to cDNA. cDNA was used as a template in qPCR assays targeting INF-γ and β actin mRNA. The relative mRNA expression was statistically analyzed using the Kruskal–Wallis test, which was followed by Dunn’s multiple comparison test. The statistical significance is indicated as (* = significant at *p* < 0.05).

The mRNA expression of IFN-γ in the lungs, kidneys, and reproductive tissues (magnum, isthmus, and uterus) in vaccinated and mock vaccinated chickens was quantified using real-time qPCR technique and illustrated in Figure 5. In the lungs, the mRNA expression of IFN-γ was higher (*p* < 0.05) in chickens that received vaccination strategies 1 and 2 when compared to mock vaccinated chickens (Figure 5a). In the kidneys, the mRNA expression of IFN-γ was higher (*p* < 0.05) in chickens that received vaccination strategy 2 when compared to mock vaccinated chickens (Figure 5b). In the reproductive tissues, we observed a significantly higher mRNA (*p* < 0.05) expression of the IFN-γ only in the magnum of the chickens that received vaccination strategy 2 when compared to the mock vaccinated controls (Figure 5c–e).

## 4. Discussion

Depending on the IBV strain, lung, kidney and reproductive tract tissue functions can be impacted [5,6,7,8]. This prompted us to examine whether two commonly used IB vaccination strategies in Canadian layer operations systemically induce an adequate immune response in various body systems. Our findings are three-fold. First, we found that vaccination strategy 2, which included the administration of inactivated IB vaccine following multiple live attenuated vaccinations, induced significantly higher serum anti-IBV antibody responses compared to the mock vaccinated controls. The higher serum anti-IBV antibody titers induced by vaccination strategy 2 had a higher neutralizing ability against IBV Mass and DMV/1639 variants when compared to that induced by vaccination strategy 1. Although the vaccination strategy 2 induced systemic antibody responses, neither vaccination strategies increased CD4+ and CD8+ T cells in the spleen. Second, both vaccination strategies recruited CD4+ T cells into the examined tissues except in the kidneys, magnum, and uterus, as well as CD8+ T cell into all examined tissues, except for the uterus. Third, both vaccination strategies induced significant IFN-γ mRNA expressions only in the lungs. In the kidneys and magnum, only vaccination strategy 2 induced a significant IFN-γ mRNA expression.

Previously, it was shown that pullets vaccinated with live attenuated vaccines followed by inactivated vaccines given prior to the lay attained protection against challenges acquired during the laying period (38 weeks) with M41 IBV [32]. Similarly, vaccination strategies with combinations of live attenuated and inactivated vaccines have also provided protection against the reduction in egg production, quality issues, and renal tissue damage induced by the IBV D1466 infection [30,31]. The addition of an inactivated IB vaccine in vaccination programs induced a better anti-IBV antibody response in serum than live attenuated vaccines given alone [30], which agrees with our serum anti-IBV antibody data. In our study, a serum anti-IBV antibody response was assessed at two different time points (3 weeks and 10 weeks post last vaccination). Vaccination strategy 2 induced higher anti-IBV antibody concentrations compared to vaccination strategy 1 at two time points. Therefore, a consistently higher antibody response was detected 10 weeks post last vaccination in our study. The persistent serum anti-IBV antibody response may be explained by the fact that inactivated vaccines are also developed with oil adjuvants that increase antigen persistence [41]. Since hens are not vaccinated during the laying period, a persistent anti-IBV antibody response is an advantage, which could potentially give laying hens longer protection against the IBV infection as has been indicated by our serum neutralization data. Previously, it has been shown that a vaccine-induced immune response is serotype specific [16] and in agreement with this, we also observed that the serum neutralization titer of serum obtained by vaccination strategy 2 was higher against the IBV Mass serotype than against IBV DMV/1639. It is important to note that our tested vaccination strategies did not include the IBV DMV/1639 serotype but the IBV Mass serotype.

Although a serum anti-IBV antibody response was evident after employing vaccination strategy 2, changes in CD4+ and CD8+ T cells in the spleen following vaccination were not observed. It has been previously shown that CD4+ and CD8+ T cell responses are strong between 8 and 14 days following an IBV infection [42], and it is possible that 10 weeks post last vaccination was not the correct time point to examine T cell responses in the spleen.

It has been shown that both CD4+ and CD8+ T cells specific to IBV are involved in the clearance of IBV with CD8+ T cells playing a critical role [43,44]. Although we did not investigate if CD4+ and CD8+ T cells are IBV specific, we observed increased recruitment of both these cells in the examined tissues. Since vaccine antigens can be delivered to the respiratory tract, kidney and oviduct, it is possible that the T cell subsets we detected in these organs were antigen-specific since, in a different context, it has been shown that the antigen-specific T cells accumulate preferentially within the site containing the specific antigen [45]. This shows that the organ-specific recruitment of these T cell subsets may be used as a surrogate measurement for the antigen-specific T cell response. In the lungs, we observed a higher recruitment of CD4+ and CD8+ T cells by both vaccination strategies. This was not surprising since the live attenuated vaccines were given via the ocular route, which allows the vaccine to route to the respiratory tract. Previously, it has been shown that live attenuated vaccines administered via the ocular route induce strong cell-mediated immune responses in respiratory tissues [46]. The vaccines given onto the conjunctiva can be disseminated to target organs such as the kidneys and the reproductive tract similar to a wild type IBV but at a reduced and slower rate [35]. This allows the induction of host responses in faraway mucosal surfaces as we observed recruitment of both CD4+ and CD8+ T cells in these tissues following the administration of both vaccination strategies.

IFN-γ is an important cytokine associated with the cell-mediated immune response and it is known to be produced by activated T cells [47], among other immune cells (i.e., natural killer cells and macrophages) [47]. Based on our observations, IFN-γ mRNA expression was higher in the lungs, kidneys, and magnum in chickens who received vaccination strategy 2 and not vaccination strategy 1. In agreement with our findings, increased INF-γ mRNA expression in respiratory tissues following IB vaccination at the day of age has been previously shown [48].

Although we investigated the immune response induced by two vaccination strategies practiced by the layer industry in Canada, we did not investigate the mechanisms involved in the difference in the immune response induced by these two vaccination strategies. In this study, we used commercially available IB live attenuated and inactivated vaccines, and we could not obtain the information regarding the amount of antigen delivered to animals by live attenuated and inactivated vaccines. The difference between vaccination strategies 1 and 2 is the last vaccination; group 1 had a live attenuated vaccine and group 2 had an inactivated vaccine. In addition to the difference in the antigen amount, the inclusion of an adjuvant in the inactivated vaccine and the use of a parenteral route may have contributed to the higher immune response generated by vaccination strategy 2. However, this needs further investigation.

## 5. Conclusions

Our findings suggest that pullets should be vaccinated with an inactivated vaccine primed with live vaccines in order to induce a better immune response characterized by antibody- and cell-mediated immune responses. The serum neutralization assay data confirmed that the vaccination strategy 2 serum (a combination of live attenuated and inactivated vaccines) is more effective at neutralizing the IBV Mass and DMV/1639 variants than the vaccination strategy 1 serum. Further, immune responses in mucosal tissues such as the lungs, kidneys, and the reproductive tract are stronger when chickens receive a combination of live attenuated and inactivated vaccines at least in the magnum, which potentially may curtail IBV-induced clinical manifestations in multiple body systems. Further studies are required to investigate if the immune response induced by vaccination strategy 2 is protective against IB induced by emerging IBV variants.

## Figures and Tables

**Table 1:** Experimental design indicating the vaccination strategies used. Live attenuated infectious bronchitis (IB) vaccines were administered via eye drops, whereas the inactivated IB vaccine was given intramuscularly. X = vaccinated.

Group	Live Attenuated Vaccine					Inactivated Vaccine
	Mass 3 Weeks	Mass + Conn 5 Weeks	Mass 8 Weeks	Mass 12 Weeks	Mass 16 Weeks	Mass 16 Weeks
Group 1 (*n* = 5)	X	X	X	X	X	
Group 2 (*n* = 7)	X	X	X	X		X
Group 3 (*n* = 5)						

## Data Availability

The datasets used and/or analyzed within the frame of the study can be provided by the corresponding author upon reasonable request.

## Supplementary Materials

The following are available online at www.mdpi.com/xxx/s1/, Figure S1: Representative immunofluorescent images of CD4+ T cells; Figure S2: Representative immunofluorescent images of CD8+ T cells.
