# Supplementary material for: Immune Responses in Laying Hens after an Infectious Bronchitis Vaccination of Pullets: A Comparison of Two Vaccination Strategies
Source: Vaccines (Basel). 2021 May 20;9(5):531. doi: 10.3390/vaccines9050531 (PMC8161194; doi:10.3390/vaccines9050531)
Supplement: Supplementary file 1 [file vaccines-09-00531-s001.zip › vaccines-1127934-supplementary.pdf]

vaccines

MDPI

Article

Immune responses in laying hens after an infectious bronchitis vaccination of pullets: A comparison of two vaccination strategies

Sabrina M. Buharideen [1], Mohamed S. H. Hassan [1], Shahnas M. Najimudeen [1], Dongyan Niu [1], Markus Czub [1], Susantha Gomis[2], and Mohamed Faizal Abdul-Careem[1]*

Figure S1: Representative immunofluorescent images of CD4+ T cells

a (Lungs CD4+ T cells)

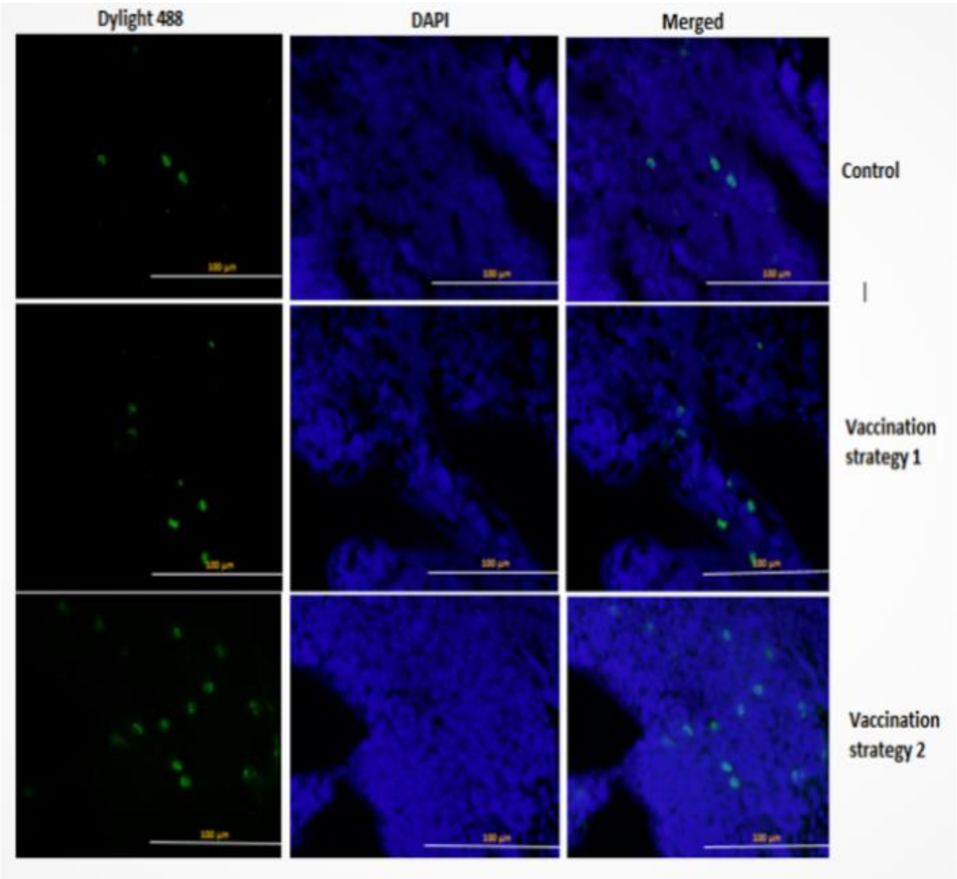

b (Kidney CD4+ T cells)

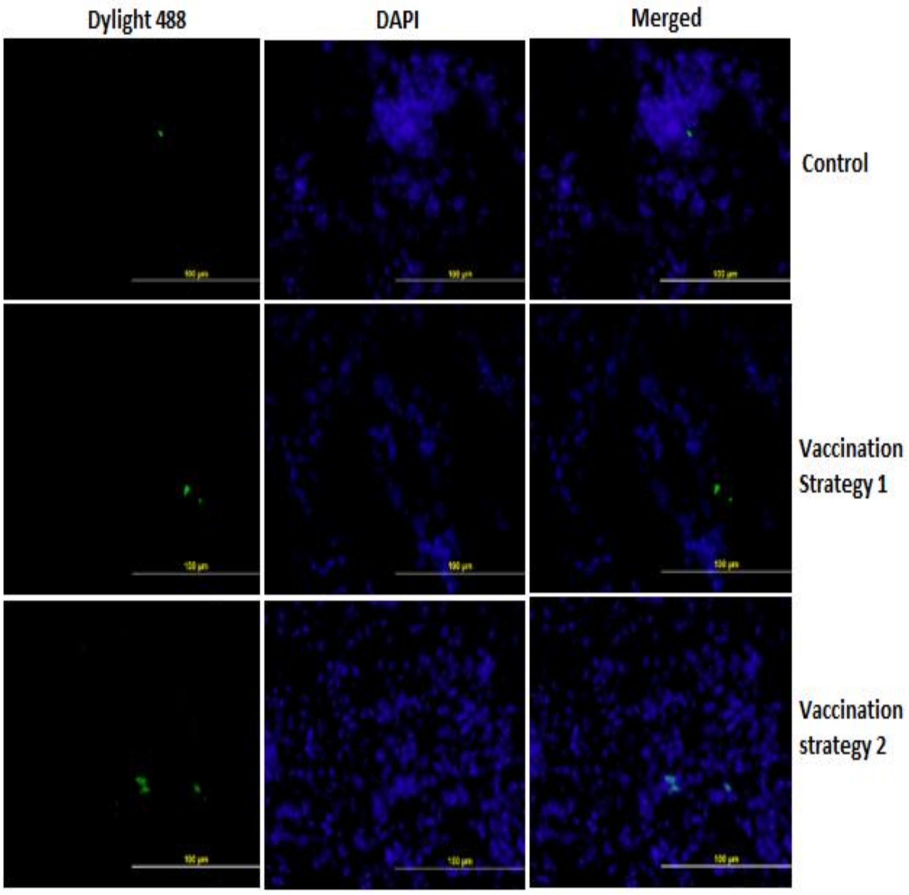

c (Magnum CD4+ T cells)

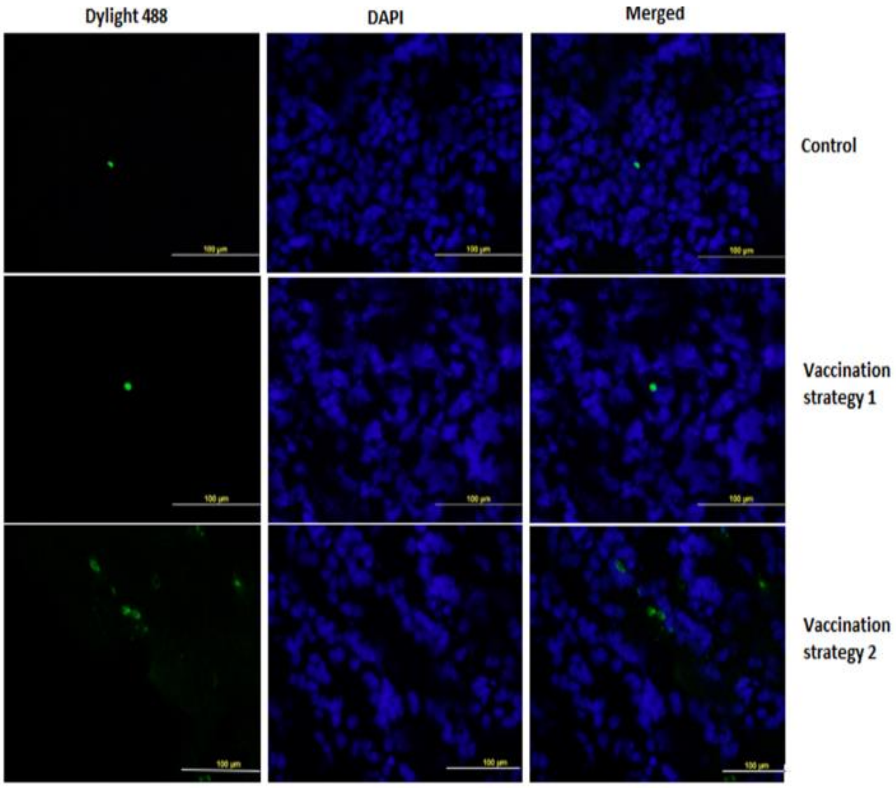

d (Isthmus CD4+ T cells)

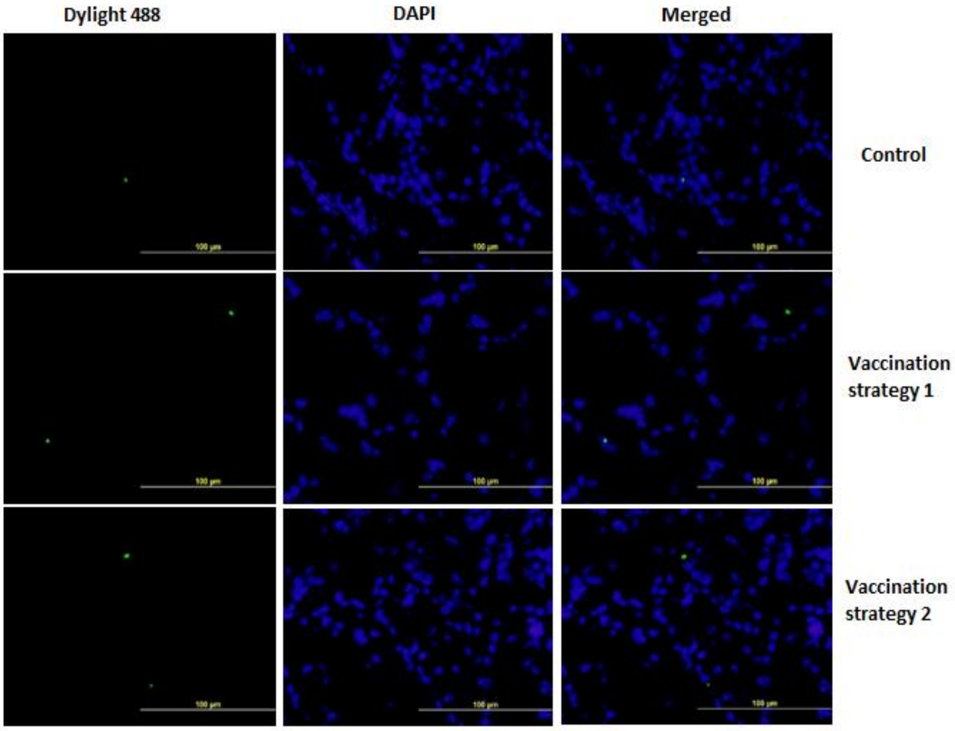

e (Uterus CD4+ T cells)

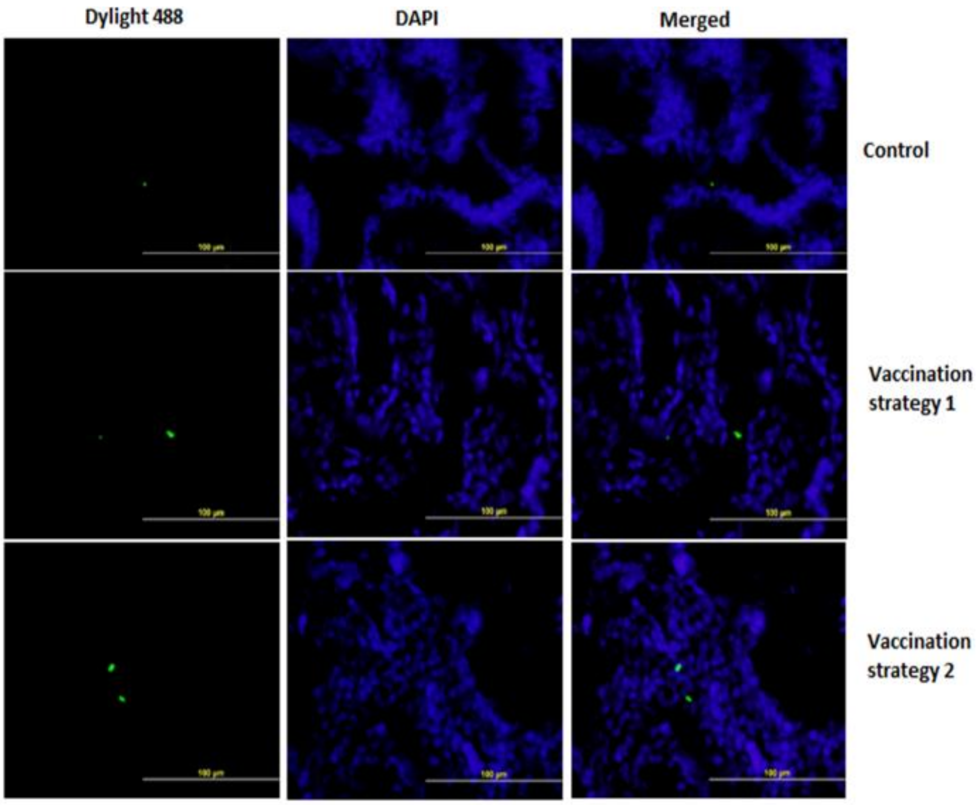

Supplementary Figure 1. Representative immunofluorescent images of CD4+ T cells in lungs (a), kidneys (b), magnum (c), isthmus (d) and uterus (e). The cryopreserved tissues belong to the chickens that received vaccination strategy 1 and 2 and mock vaccinated chickens were sectioned, immunoassayed using monoclonal antibodies directed against chicken CD4. The mounting media contained the nuclear stain, The CD4 positive signals (green) and nuclear stained areas (blue) were captured in order to merge the images.

Figure S1: Representative immunofluorescent images of CD8+ T cells

a (Lungs CD8+ T cells)

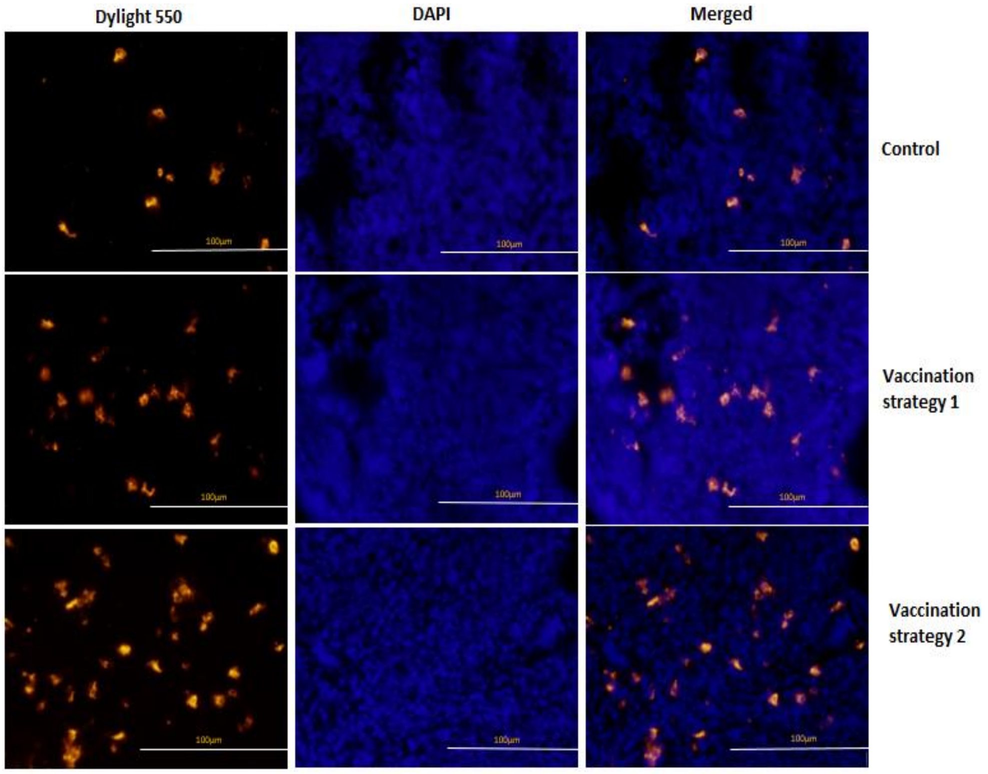

b (Kidneys CD8+ T cells)

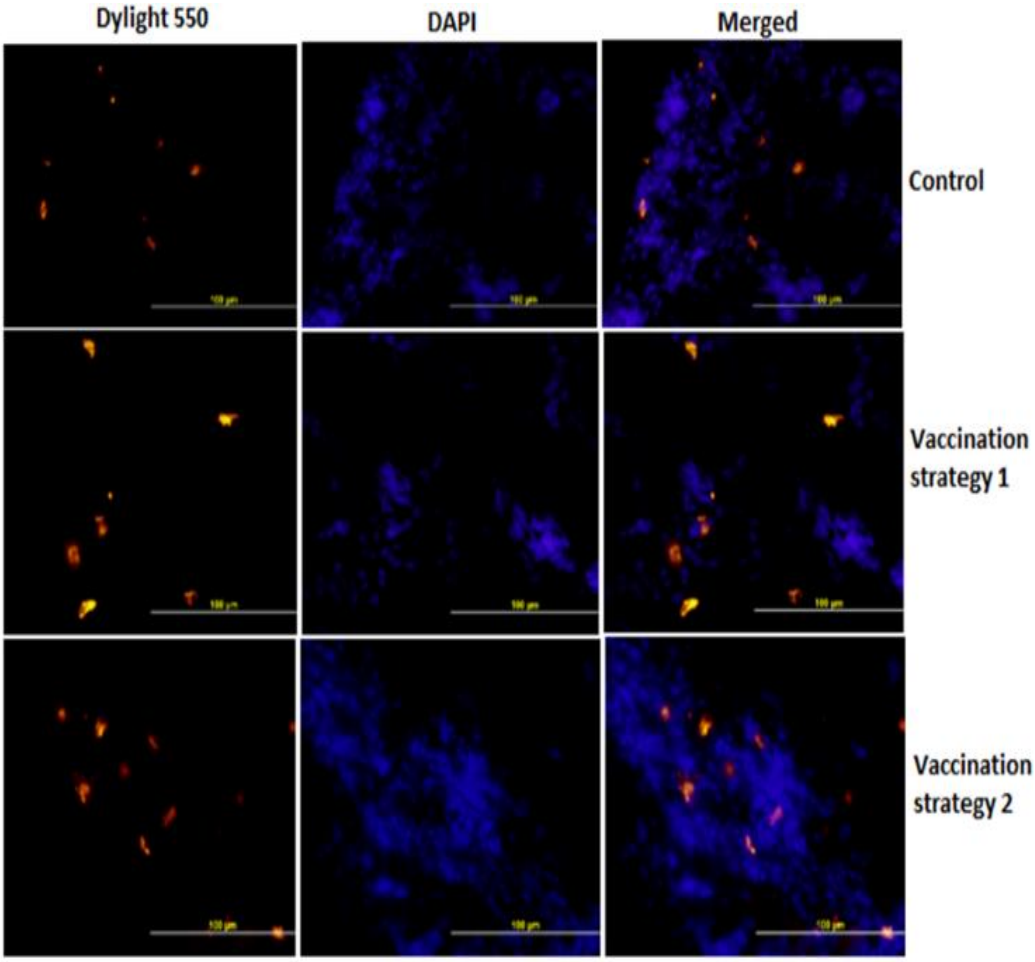

c (Magnum CD8+ T cells)

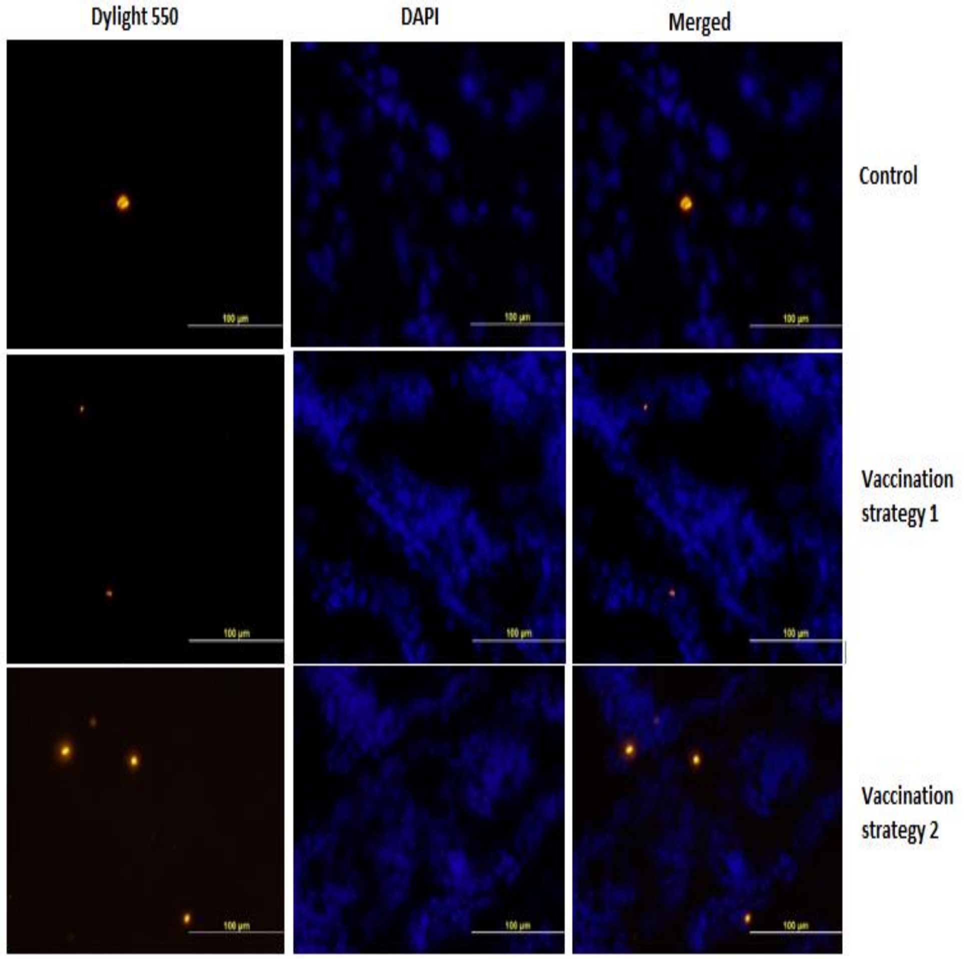

d (Isthmus CD8+ T cells)

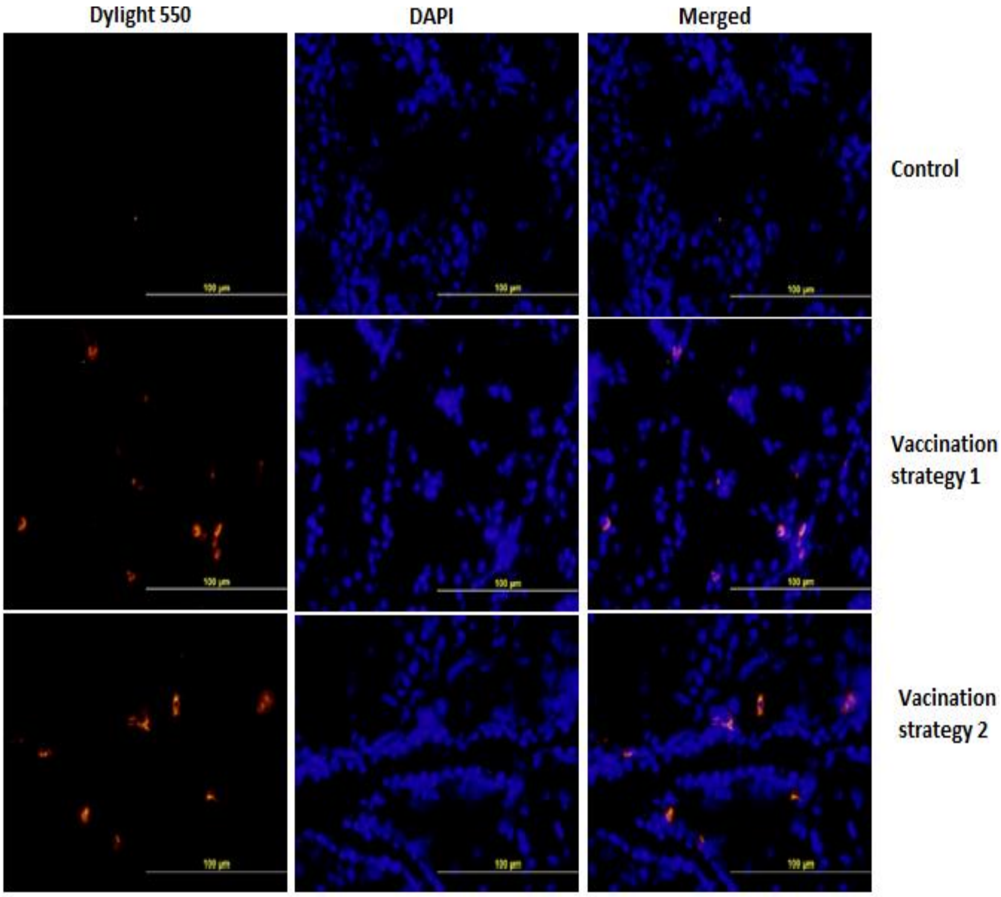
Dylight 550
DAPI
Merged
Control
Vaccination strategy 1
Vacination strategy 2
100 μm

e (Uterus CD8+ T cells)

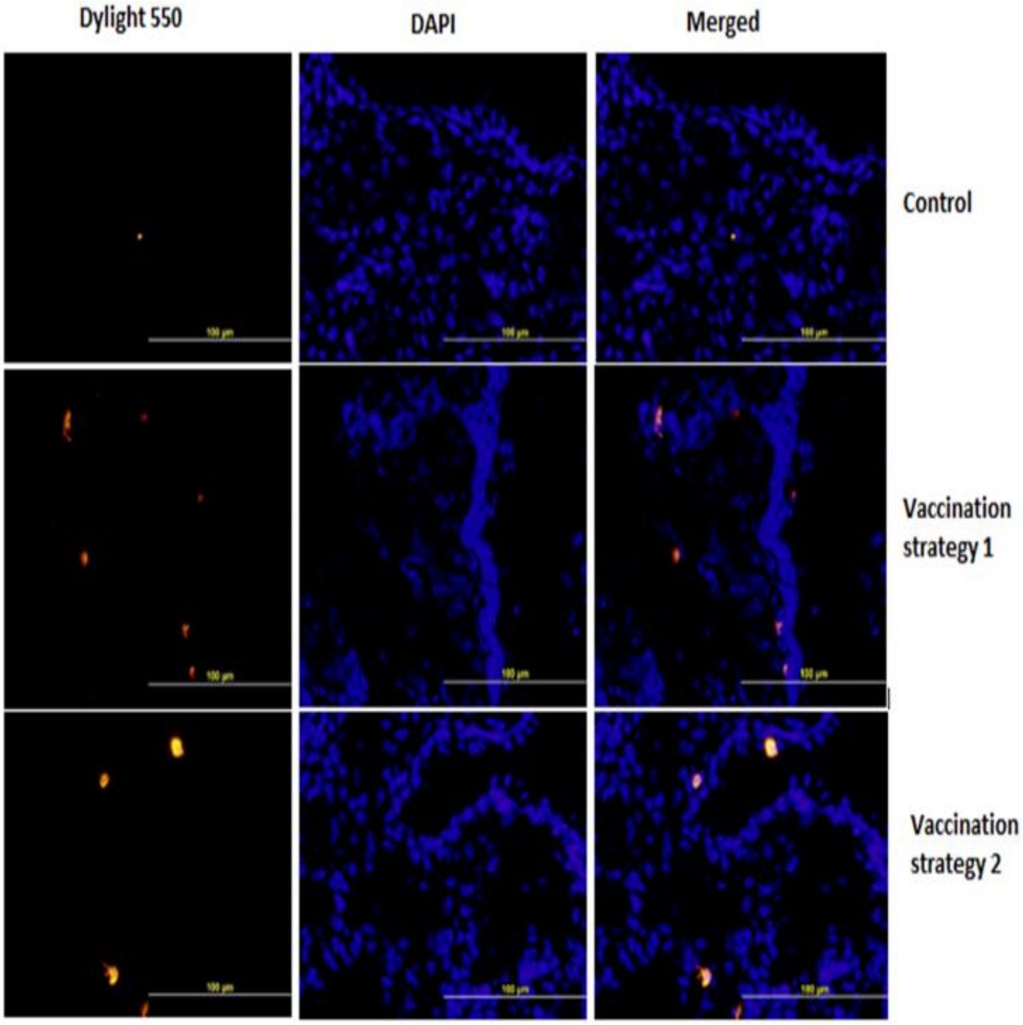

Supplementary Figure 2. Representative immunofluorescent images of CD8+ T cells in lungs (a), kidneys (b), magnum (c), isthmus (d) and uterus (e). The cryopreserved tissues belong to the chickens that received vaccination strategy 1 and 2 and mock vaccinated chickens were sectioned, immunoassayed using monoclonal antibodies directed against chicken CD8. The mounting media contained the nuclear stain, The CD4 positive signals (green) and nuclear stained areas (blue) were captured in order to merge the images.
